# Experience of Percutaneous Closure of Ventricular Septal Defects in 140 Patients With Different Duct Occluders in a Tertiary Care Rural Hospital in Central India

**DOI:** 10.7759/cureus.42591

**Published:** 2023-07-28

**Authors:** Benumadhab Ghosh, Isha Sahai, Gajendra Agrawal, Satish Khadase, Tarun Rao, Akash Lohakare, Anuj Chaturvedi, Shantanu Gomase

**Affiliations:** 1 Cardiology, Jawaharlal Nehru Medical College, Datta Meghe Institute of Higher Education and Research, Wardha, IND; 2 Pediatrics, Jawaharlal Nehru Medical College, Datta Meghe Institute of Higher Education and Research, Wardha, IND

**Keywords:** muscular vsd, perimembranous, cocoon ductal device, lifetech ductal device, amplatzer duct occluder, percutaneous coronary intervention, ventricular septal defect closure

## Abstract

Background

Ventricular septal defects (VSDs) are the most common type of septal defects in early infants and are very complicated. This has paved the way for the development of new minimally invasive procedures for interventional cardiologists. This study presents our experience using duct occluders instead of conventional ventricular septal devices in the Department of Cardiology at Acharya Vinoba Bhave Rural Hospital (AVBRH) in central rural India. This study aimed to review success and complications and assess safety and its relation to age, sex, size of the VSDs, type of VSD, and types of devices used after transcatheter closure of perimembranous and muscular VSDs using various types of duct occluders.

Methodology

This retrospective study included patients who underwent percutaneous VSD device closure at the AVBRH between July 2017 and December 2020. We reviewed the patients' medical records to recognize imaging, clinical, and interventional data pre- and post-procedure and at the last follow-up.

Results

The success rate of VSD closure was 98.6%, one (0.7%) out of 81 females developed a complication due to device dislodgement, and one male aged six years (0.7%) out of 59 developed a post-procedural complication; hence, the total failure rate was 1.4%. The perimembranous type had no complication, and the muscular type had two (14.3%) unsuccessful procedures.

Conclusion

This study has concluded an impressive percentage of VSD closure, showing no mortality and low morbidity, using a percutaneous approach with different duct occluders. As the type of device used is not correlated with device failure and failure rate, duct occluders will be financially helpful in the closure of VSD in indicated patients.

## Introduction

Ventricular septal defects (VSDs) are due to a congenital malformation or stoppage of the formation of the interventricular septum during intrauterine embryological heart development. VSD is the most common congenital heart disease (CHD) in children. It has been observed that 37% of all pediatric congenital heart disease (CHD) cases are VSDs [1]. An incidence of 0.3% is seen in newborns [1]. VSDs are complex and classified into four major groups: perimembranous, muscular, infundibular, and atrioventricular types [1]. Several complications, such as pulmonary arterial hypertension, ventricular dysfunction, heart failure, and arrhythmias, can be averted by surgeons if these VSDs are closed as early as possible [2]. Traditionally, surgery has been the original mode of treatment for closing VSDs [3-6]. However, several complications have been observed, such as scarring, sternotomy, discomfort, and, most importantly, mortality and morbidity [5,6]. As VSDs are complex in early infancy, new minimally invasive procedures have been developed by collaborating interventional cardiologists with cardiac surgeons [7]. Over the last decade, the percutaneous approach has received special attention from interventional cardiologists owing to its positive outcomes [8-10]. Closure of VSDs via the transcatheter approach using a device is an alternate method to surgery in some anatomical types of VSDs. Recent developments in percutaneous closure of devices have the major precedence of remarkably reducing a patient's duration of hospital stay [11].

This study aimed to review our experiences in the closure of VSDs by a percutaneous approach using duct occluders for perimembranous and multifunctional duct occluders for muscular, and its initial results by minimally invasive procedures and two years follow-up for these patients at the Cardiology Department of Acharya Vinoba Bhave Rural Hospital (AVBRH) in Wardha, India. Here, we present our experience using duct occluders instead of conventional ventricular septal devices.

## Materials and methods

This retrospective study included all patients who received percutaneous VSD device closure at the AVBRH from July 2017 to December 2020. Transthoracic echocardiography (TTE), electrocardiogram (ECG), chest X-ray, and blood tests for liver function (LFT), kidney function (KFT), and blood investigations such as prothrombin time, activated partial thromboplastin time (APTT), and platelet count were performed before the procedure to identify any bleeding disorders. Prior to the procedure, the presence of aortic regurgitation (AR), tricuspid regurgitation (TR), or mitral regurgitation (MR) was ruled out. During the procedure, we recorded the fluoroscopy time. The primary indication for selecting patients for the VSD closure is that the Qp/Qs is more than 2:1. Aortic right coronary cusp (RCC) prolapse patients were not indicated for the transcatheter approach. After the procedure, immediate echocardiography, follow-up ECG, and the presence of any complications were reviewed and noted.

After reviewing the medical records for clinical evaluation of the entire data and a thorough review of electrocardiograms (ECG) and transthoracic electrocardiograms (TTEs) in pre- and post-procedural visits and follow-ups, we searched the database to identify patients who had undergone transcatheter VSD closure. We analyze cardiac catheterization and angiogram reports to understand the procedures' characteristics and hemodynamics. Pulmonary hypertension is an essential factor that we had to rule out for the selection of patients for the patch closure. Patients having mild to moderate pulmonary hypertension (<50 mmHg) were indicated for the intervention. The size of the defects was assessed using TTE and left ventricular (LV) angiogram. Patients in whom the aortic rim was less than 2 mm were only indicated in the procedure. Patients with aortic rim over 2 mm were not indicated for the procedure. The selection of device size was according to the size of the defect, as mentioned in the results section. The devices used in the patients were selected based on stock availability in the institute, as they have similar characteristics and purposes. This was done to obtain true results of the devices as they have similar functions. Cardiac interventions were performed under sedation, thus avoiding post-procedural complications of anesthesia via the transfemoral and transjugular approaches under TTE guidance and LV angiogram. The transjugular approach was performed only in those patients who had muscular VSDs. In patients 1-10 years of age, in whom the transjugular approach was performed under sedation, an ultrasound-guided catheter was used to perform the intervention to prevent complications such as carotid puncture and hematoma. After the procedure, follow-ups were performed immediately, at three and six months and at one year and two years. A procedure is considered successful if a stable device is deployed correctly across the septal defect without any intricacy to adjoining structures and without any residual shunt.

Intervention

The procedure was performed via the right internal jugular or femoral route by sedating the patient. Most VSDs were performed by crossing the left ventricle (LV) to the right ventricle (RV) using a Judkins right catheter and Terumo wire (Terumo Corporation, Shibuya City, Tokyo, Japan). The wire was sent through the left or right pulmonary artery (LPA/RPA) and then snared out using a gooseneck snare from the other end (venous end) by making a loop, which is an arteriovenous (AV) loop. The delivery sheath or guiding catheter was approached around the wire from the venous end, thus covering the guide wire (kissing technique). The tip of the guide catheter reached the aortic arch. Subsequently, under the guidance of TTE and fluoroscopy, the device was placed using a delivery sheath. In a few cases, the tip of the guiding catheter was placed in the LV, and the VSD was extended from the RV side via the right coronary artery (RCA) guiding catheter. LV angiography was performed after deployment to check for any residual shunts.

Statistical analyses in our study were done with the help of Statistical Package for the Social Sciences (SPSS) for Windows version 25.0 (IBM SPSS Statistics, Armonk, NY, USA). The mean and interquartile range were used to show continuous variables. The frequency and percentage of data are shown categorically. We analyzed the procedure results and risk factors. The success of all procedures was investigated using outcome variables, along with the incidence of all post-procedural complications. We included some independent variables in the investigation: the patient's age at the time of the procedure and sex. We also incorporated the diameter of the defect, as calculated on TTE, and screening for ventricular septal aneurysm, AR, TR, and MR was performed. Binary logistic regression was used for univariate analysis if the risk factors were used for univariate analysis. The Institutional Ethics Committee (IEC) of Jawaharlal Nehru Medical College, Datta Meghe Institute of Higher Education and Research approved the study.

## Results

This study included 140 patients: 81 (57.9%) were females, and 59 (42.1%) were males. A total of 140 percutaneous procedures (no patient underwent two device placements) were performed between July 2017 and December 2020 for the closure of a septal defect (VSD) at a mean age of 8.4 years (range: 1-30 years). Data were collected retrospectively from hospital records. Perimembranous VSD was the most common among the patients. The procedure was indicated in patients whose shunt Qp/Qs was more than 2:1.

The closure was performed using an occluder with a mean diameter of 6/8 mm via the transfemoral approach in 126 (90%) patients and the jugular venous approach in the remaining 14 (10%) patients. The mean diameter of the defects, as determined by transthoracic echocardiography (TTE) and LV angiogram, was approximately 6.5±2 mm (n=140). Post-procedural echocardiography showed no residual shunt, and the device was well-placed in all patients who underwent the procedure. We noted no prior aortic regurgitation. Mild mitral valve regurgitation was observed in 10% of the patients. The mean duration of hospital stay was 3-5 days for complication-free patients and 5-7 days for patients with complications during or after the procedure (Table 1). None of the deaths were related to the procedure. Different parameters were considered for the study population, as summarized in Table 1.

**Table 1:** Characteristics of the study population (N=140 procedures) The table depicts various parameters that have been considered for finding out the total success and failure rates. VSD: ventricular septal defect

Parameters	Total procedures (N=140)	Successful procedures	Unsuccessful procedures	Chi-square	p-value
Mean age (years)	8.4 (1-30)		5.5		
Gender				0.0499	0.8233
Female	81 (57.9%)	80 (98.7%)	1 (1.2%)		
Male	59 (42.1%)	58 (98.3%)	1 (1.7%)		
Age (years)				0.6889	07086
1-10	104 (74.3%)	102 (98.07)	2 (1.9%)		
10-20	32 (22.8%)	32 (22.8%)	0 (0%)		
20-30	4 (2.9%)	4 (2.9%)	0 (0%)		
VSD type				15.975	<0.001
Perimembranous	126 (90%)	126 (90%)	0 (0%)		
Muscular	14 (10%)	12	2 (14.3%)		
Average hospital stay	-	3-5 days	5-7 days		

Two variants of VSD (perimembranous and muscular) were treated using seven types of devices: Amplatzer ductal device, Cocoon, LifeTech Cera, LifeTech HeartR, LifeTech KONAR MF, Occlunix, and Occlutech (Tables 2, 3). We observed the success and failure rates of the devices most commonly used in VSD closure (Tables 2, 3).

**Table 2:** Types of VSD The table concludes that Cocoon has been used most frequently for the closure of VSD. VSD: ventricular septal defect

VSD devices	Total	Successful	Unsuccessful
Amplatzer ductal device	3	3	0
Cocoon	99	99	0
LifeTech Cera	7	7	0
LifeTech HeartR	10	10	0
LifeTech KONAR MF	12	10	2
Occlunix	8	8	0
Occlutech	1	1	0

**Table 3:** Devices in various types of VSD closure The table concludes that the devices used in the closure of muscular VSD showed more unsuccessful events as compared to the devices used in the closure of perimembranous VSD. VSD: ventricular septal defect

Perimembranous	Successful	Unsuccessful	Total	Chi-square	p-value
Cocoon	99	0	99 (70.71%)		
Amplatzer Perimembranous	2	0	2 (1.42%)		
LifeTech Perimembranous	17	0	17 (12.14%)		
Occlunix	8	0	8 (5.71%)		
Occlutech	1	0	1 (0.71%)		
Muscular	Successful	Unsuccessful	Total	Chi-square	p-value
LifeTech Muscular	10	2	12 (85.71%)	0.197	0.6572
Amplatzer Muscular	1	0	1 (0.71%)	0.197	0.6572

Cocoon was used most commonly (70.71%) of the total devices used, with a 0% failure rate. LifeTech Muscular was used in 12 (85.71%) procedures, and two unsuccessful procedures were associated with the device. Among the 140 patients, we successfully deployed 138 (98.6%) devices during 140 interventions. Out of 59 males, one patient developed a transient complete heart block (CHB) during the procedure and reverted to sinus rhythm immediately, and the device was then successfully deployed. Of the 81 females, one had device dislodgement into the aorta, which was retrieved successfully and sent for surgery. Complications were noted in a six-year-old symptomatic male who suffered a complete heart block and underwent the procedure using the LifeTech Multifunctional Occluder (LT-MFO) multipurpose device. The other complication, device dislodgement into the aorta, occurred in a five-year-old female, where the device used was LT-MFO. In these patients, the procedure was performed using a jugular approach. Interventions for these complications are shown in Tables 4, 5.

**Table 4:** Complications following the percutaneous VSD closure The table shows that muscular VSD had two unsuccessful procedures, and the device used was LT-MFO. VSD: ventricular septal defect, LT: LifeTech, MFO: Multifunctional Occluder, CHB: complete heart block, ADO: Amplatzer Duct Occluder

Number	Age (years)	Gender	VSD type	Device type	Complication	Outcome
1	6	Male	Muscular VSD	LT-MFO-8-6	Femoral artery occluded with thrombus, CHB detected during the procedure	Second device placed (ADO)
2	5	Female	Muscular VSD	LT-MFO-7-5	Device dislodgement into the aorta	Surgically retrieved immediately

**Table 5:** Univariate analysis of risk factors for complications following percutaneous VSD closure The table shows the risk factors for the failure of procedures. VSD: ventricular septal defect, TTE: transthoracic echocardiography

Variable	Univariate	Univariate
	Early complications present	Early complications absent
	2	138
Mean age	5.5 years	8.4 years
Age 1-10 years		
Yes	2	-
No	0	-
Gender		
Male	1	58
Female	1	80
Size of VSD via TTE	7.5 mm	6.5±2 mm

The transfemoral and transjugular approaches were used to close the defects. Arteriovenous (AV) looping was performed in 121 (86.4%) procedures, whereas closure was performed by crossing the VSD from the RV side without making an AV loop in 19 (13.6%) procedures (Table 6). In the first six months, 129 (92.14%) of 140 patients showed up for follow-up. In the first year, 125 (89.29%) patients appeared, and at the end of two years, 107 (76.42%) patients came for regular follow-up (Table 7).

**Table 6:** Data on the procedures performed The table shows that arteriovenous looping has been done in the majority of patients. VSD: ventricular septal defect, RV: right ventricle, AV: arteriovenous, TTE: transthoracic echocardiography

Variable	Number (%)
Looping	
Arteriovenous looping	121 (86.4%)
Crossing VSD from RV side, not AV looping	19 (13.6%)
Procedure	
Qp/Qs	Shunt >2:1
VSD diameter via TTE (mm)	6.5±2 mm
Devices	
Occluder	127 (90.7%)
Multipurpose device	13 (9.3%)
Device size (mm)	6.5±2

**Table 7:** Follow-up record summary The table shows that out of 140 patients, 107 were compliant for two years of follow-ups.

6 months (n=140)	1 year (n=140)	2 years (n=140)
129 (92.14%)	125 (89.29%)	107 (76.42%)

**Figure 1:**
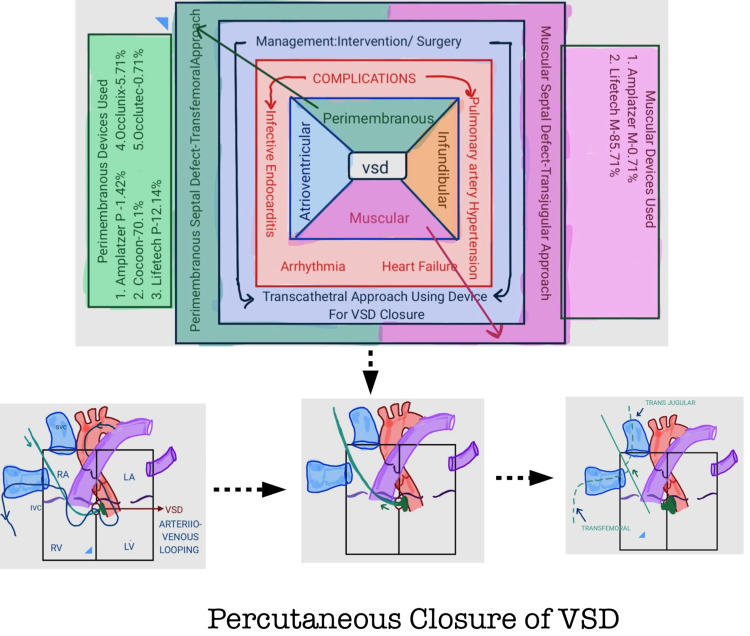
Percutaneous closure of VSD VSD: ventricular septal defect

After a complete analysis of the risk factors, we concluded that the age group between one and 10 years could be a risk factor for the failure of the procedure. The mean age for successful VSD closure procedures was 8.4 years and 5.5 years (p≤0.01) for unsuccessful procedures. However, there was no significant association between the probability of device failure and age (p=0.7086), sex (p=0.8233), and device type (p=0.6572). There was a significant association between VSD type and device failure (p≤0.001). We noted that the muscular type of VSD had two (14.3%) unsuccessful procedural events; on the other hand, the perimembranous type had no such complications. This makes us consider the use of duct occluders for the closure of the perimembranous type of VSD. The success rate of VSD closure was 98.6%, with one (0.7%) out of 81 females due to device dislodgement and another was one male aged six years (0.7%) out of 59, with a post-procedural complication, with a total failure rate of 1.4%. We have summarized the methodology and results in Figure 1.

## Discussion

In this study, we report our evaluation of treating VSDs of various types using different kinds of devices and any postsurgical complications. However, previous studies have reported positive results [12-14]. The prediction of complications before the procedure is complicated. We assessed the cases starting before the intervention and continuing until the follow-ups to understand the results and whether using alternate devices such as patent ductus arteriosus (PDA) devices in the VSD position was feasible. This study aimed to provide a different and successful solution for children who are lagging in growth and have low body weight and patients suffering from consequent abnormal conditions requiring multiple interventions with a low-cost budget and effective plan. We decided to follow the therapeutic procedure after thorough training and comprehensive preparation.

Intraoperative periventricular device closure was first successfully performed on a beating heart in an infant in 1998 [15]. In the studies by Holzer et al. [16] and Butera et al. [17], high success rates in percutaneous VSD closure were observed at approximately 93% and 96%, respectively. Another recent study by Al Senaidi et al. [18] found a 94.1% success rate for closure. The benefit of such a method is closing the defect directly and lowering the chances of the device falling. The chances of hemodynamic fluctuations also decreased, and corrections were made if there were any other defects.

In VSD closure, the percutaneous approach is challenging for younger patients. In addition, choosing the appropriate devices and best procedure is complex because there are no discreet guidelines. Several factors depend on the selection of the right strategy for managing VSDs. Surgical interventions are more often performed in infants with low body weights. Diab et al. [19] revealed several infants aged less than a year, including the smallest infant who underwent a percutaneous approach weighing 3.8 kg. In another study, a symptomatic male patient weighing 3.5 kg and aged three months had an uneventful mid-muscular VSD closure after surgery using an Amplatzer Muscular VSD (AMVSD) occluder (10 mm) [18]. In our study, two patients who were one year old and weighed 4.1 kg had successful VSD closure using a duct occluder. The follow-up had no early postoperative complications, complaints, or discrepancies. Holzer et al. [16] reported that patients weighing <10 kg showed a relatively increased occurrence (58.3%) of adverse events compared to patients weighing >10 kg (25%) (p=0.0285). Thus, interventional cardiologists must be cautious in selecting device sizes and types for small infants while conducting VSDs.

To achieve safer VSD closures, the proper selection of devices is essential [20]. Currently, researchers select devices that predominantly depend on the VSD morphology. Preferences are given to ductal devices for the upper muscular or membranous sites. Muscular VSD devices are used in muscular types of VSDs. In most cases, if the aneurysm is large, the device is placed within the aneurysm to avoid encroachment on the aortic valve. Furthermore, devices with smaller right ventricular disc sizes, such as duct occluders, are used to prevent entrapping of the tricuspid valve. However, limiting factors, such as the aneurysm's strength and number of apertures, should be ruled out. In our study, however, there was no aneurysm that would anticipate the success of the procedure or any complications after the procedure.

Although there were no deaths, we observed early complications in two (1.42%) patients. In our study, fewer patients had large defects. Butera et al. [21] reported that 12 patients experienced 13 significant early complications (11.5%). Among them, two were embolization of the device. In this study, we observed no variables that would allow us to predict early complications.

CHB is a well-known condition relating to complications of perimembranous VSD closure. One of the critical early complications associated with the closure of perimembranous VSD is CHB. In the current study, only one patient developed such complications (0.71%). Other studies, such as those of Holzer et al. [16] and Arora et al. [22], have reported 2% and 1.9% rates, respectively. The device can cause direct mechanical injury during the procedure, leading to heart block; in contrast, edema, local inflammation, and fibrosis may occur after the procedure is activated by ventricular retention discs [23]. In our study, the proportion was very low, and the patient had average body weight with a comparatively larger device using two discs. In this study, we believe the lower incidence of CHB was due to using duct occluders with one disc, similar to Amplatzer Duct Occluder (ADO) type I. It is difficult to entirely avoid heart block due to anatomical conditions such as large defects with large retention discs and perimembranous VSDs. Follow-ups are highly indicated as the block in the heart can exhibit later complications after a successful intervention.

Approximately 9.2% of both aortic and tricuspid valve regurgitation was reported by Holzer et al. [16]. Still, the conditions of most of those patients were observed to have been better at the last follow-up. In our study, two patients underwent surgical device retrieval and unsuccessful closure of the VSD. This emphasizes the necessity of carefully evaluating the atrioventricular and semilunar valves before releasing the device. However, determining several complications of the valve related to the device cannot be done before the release because, after its placement, the device reconfigures itself after releasing the traction of the delivery cable [16].

Our study had a few limitations. It was retrospective, and the study population was not uniform, comprising adults and pediatric patients. Thus, the results cannot be specific to any definite age, although data from follow-ups have been reported. Furthermore, the study was conducted at a single center; thus, the results may be nonspecific to larger populations.

## Conclusions

The study has shown a good percentage of ventricular septal defect (VSD) closure rates with zero mortality using the percutaneous intervention approach with duct occluders. As the type of device used is not correlated with device failure and failure rate, using duct occluders via transcatheter procedures performed for VSD closure is economical and effective in patients from low socioeconomic strata, with unsuitable morphological features, and unfit for conventional surgery. We also observed that the procedure significantly reduced the duration of hospital stay. Follow-ups of the patients were taken up to two years; we found no complaints or complications in patients who visited the follow-up visits. The outcomes of VSDs on the cardiovascular system should be monitored for a long duration during follow-up. However, none of our patients developed disturbances in the conduction system during the years of follow-up. The duct occluders used for VSD closure served this purpose without any complications, and the conventional devices used for VSD closure are costly for patients in developing and underdeveloped countries. Hence, duct occluders can be successfully used instead of conventional VSD closure devices.
